# Oral Microbiome Using *Colocasia antiquorum* var. *esculenta* Extract Varnish in a Mouse Model with Oral Gavage of *P. gingivalis* ATCC 53978

**DOI:** 10.3390/medicina58040506

**Published:** 2022-04-01

**Authors:** Seong-Jin Shin, Seong-Hee Moon, Hyun-Jin Kim, Seung-Han Oh, Ji-Myung Bae

**Affiliations:** 1 Department of Dental Biomaterials, College of Dentistry, Wonkwang University, 460 Iksan-daero, Iksan 54538, Jeonbuk, Korea; ko2742@naver.com (S.-J.S.); shoh@wku.ac.kr (S.-H.O.); 2 Institute of Biomaterials and Implant, College of Dentistry, Wonkwang University, 460 Iksan-daero, Iksan 54538, Jeonbuk, Korea; shmoon06@gmail.com (S.-H.M.); khjin1005@wku.ac.kr (H.-J.K.); 3 Department of Oral Anatomy, College of Dentistry, Wonkwang University, 460 Iksan-daero, Iksan 54538, Jeonbuk, Korea

**Keywords:** oral microbiome, *Colocasia antiquorum* var. *esculenta*, *P. gingivalis*, varnish

## Abstract

*Background and Objective*: There is increasing interest in preventing periodontitis using natural products. The purpose of this study was to investigate the effect of *Colocasia antiquorum* var. *esculenta* (CA) varnish on the oral microbiome and alveolar bone loss in a mouse periodontitis model. *Materials and Methods*: Antibacterial activity against *Porphyromonas gingivalis* (*P. gingivalis*) ATCC 53978 and cell cytotoxicity using CCK-8 on L929 cells were measured. Balb/c mice were assigned into five groups (negative control, positive control, CA in drinking water, varnish, and CA varnish). *P. gingivalis* was administered to the mice by oral gavage three times. After sacrifice, the oral microbiome and the levels of the inflammatory cytokine IL-1β and matrix metalloproteinase-9 were analyzed. Alveolar bone loss was measured using micro-computed tomography. *Results*: CA extract showed an antibacterial effect against *P. gingivalis* (*p* < 0.05) and showed no cytotoxicity at that concentration (*p* > 0.05). Although alpha diversity of the oral microbiome did not statistically differ between the groups (*p* > 0.05), the relative abundance of dominant bacteria tended to be different between the groups. The inflammatory cytokine IL-1β was reduced in the CA varnish group (*p* < 0.05), and no difference was observed in MMP-9 expression and alveolar bone loss (*p* > 0.05). *Conclusions*: CA varnish did not affect the overall microflora and exhibited an anti-inflammatory effect, suggesting that it is possibility a suitable candidate for improving periodontitis.

## 1. Introduction

Periodontal disease is an inflammatory disease caused by bacteria and changes in the oral bacterial composition are known to cause periodontal disease [1]. Oral bacteria attached to calculus on tooth surfaces produce toxins such as lipopolysaccharides and endotoxins [2]. The toxins cause an inflammatory response in the gingival tissue, creating a countering immune response [3]. In this inflammatory immune response, inflammatory cytokines such as IL-1β and TNF-α are expressed [4] and matrix metalloproteases (MMPs) cause collagen degradation [5]. Inflammatory cytokines and MMPs can cause severe damage to the surrounding tissues [6].

The first approach to treating periodontal disease is the removal of the calculus attached to the teeth through scaling and root planning [7]. However, when the calculus is located in the deep gingival sulcus, it is difficult to remove it entirely [8]. Antibiotics and chlorhexidine can assist in the non-surgical treatment of periodontal disease but have side effects such as hepatotoxicity, renal toxicity, opportunistic infection, and discoloration [9,10]. In order to reduce these side effects, natural herb medicine must be increased [11]. Local drug delivery systems have also been developed to localize drugs in periodontal pockets [12].

*Colocasia antiquorum* var. *esculenta* (CA) is a plant of the family Tauraceae, called taro, eddoe, and is used for food and medicinal purposes [13]. CA extract is known to have antioxidant, antifungal, and antimicrobial effects [14,15]. The methanol extract of CA has exhibited antimicrobial effects against *Porphyromonas gingivalis* (*P. gingivalis*) with anti-inflammatory and anti-osteoclastogenic activities [16]. However, the effects of the ethanol extract of CA are not well known.

Several mouse models have been used to simulate periodontitis to mimic the human condition, including the ligature model, the lipopolysaccharide (LPS) injection model, and the *P. gingivalis* inoculation model [17,18,19]. The ligature model can induce alveolar bone loss quickly; however, it can cause damage to the gingival tissue due to ligation pressure [20]. The LPS injection model promotes aggressive lesions and rapid alveolar bone destruction but exhibits a different pattern than chronic periodontitis, where the alveolar bone is destroyed over an extended period [18]. This study adopted the *P. gingivalis* inoculation model, in which *P. gingivalis* was administered by oral gavage to initiate dysbiosis of oral bacteria and induce periodontitis with chronic behavior [21]. This model simulates human chronic periodontitis caused by *P. gingivalis* [22].

More than 700 microorganisms closely related with periodontitis have been detected in the oral cavity [23]. With the development of next-generation sequencing technology, it has become possible to analyze the oral microbiome, a collection of all oral microorganisms [24]. The oral microflora, oral microbiota, and more recently, the oral microbiome have all been used to describe the bacteria found in the human oral cavity [25]. Changes in the oral microbiome related to periodontitis, dental caries, and oral cancer have been investigated [26,27]; however, few papers have examined the changes in the oral microbiome by applying dental materials. If an effective agent, such as an antibiotic, alters the oral microbiome, it can cause other diseases such as candidiasis [2]. Since the oral microbiome examines the interaction of hundreds of species, in vivo tests should be done. It is crucial to analyze the oral microbiome using an animal model before clinical trials. Recently, the murine oral microbiome has been studied [28,29]. Mouse microbiome models have been the primary choice for preclinical tests for studying relationships between the microbiomes and host physiological, metabolic, immune, and neurologic phenotypes [30].

The purpose of this study was to investigate whether CA varnish effectively inhibits factors related to periodontal disease. Antibacterial effects against *P. gingivalis* and the cytotoxicity of the ethanol extract of CA were also evaluated in vitro. Using a mouse model with oral gavage of *P. gingivalis* the American Type Culture Collection (ATCC) 53978, the changes in the oral microbiome, inflammatory cytokines, and alveolar bone levels were analyzed. The null hypothesis was that the oral microbiome, alveolar bone loss, and inflammatory factor would not differ among the groups.

## 2. Materials and Methods

### 2.1. Ethanol Extract of CA

The ethanol extract of *Colocasia antiquorum* var. *esculenta* (CA) in this study was obtained from the Korea Plant Extract Bank (Korea Research Institute of Bioscience and Biotechnology, Daejeon, Korea). A stock solution (50 mg/mL) was prepared in dimethyl sulfoxide (DMSO) and stored at −20 °C before use.

### 2.2. Antibacterial Assay

To investigate the antibacterial effects of CA, *P. gingivalis* (ATCC 53978) was purchased from the American Type Culture Collection (ATCC, Manassas, VA, USA) and cultured at 37 °C in an anaerobic chamber in brain heart infusion (BHI) broth with 5 g/mL hemin and 0.5 g/mL menadione. Serial dilutions of CA were added to the 96-well plate, and *P. gingivalis* suspensions with a final concentration of 5 × 10^7^ colony-forming units (CFU) were added. The minimum inhibitory concentration (MIC) was determined as the lowest concentration of CA that inhibited the visible growth of *P. gingivalis*. For the minimal bactericidal concentration (MBC), concentrations equal to and higher than the MIC were streaked on blood agar plates and incubated for seven days. MBC was determined as the lowest concentration of CA that had no visible bacterial colonies on the blood agar plate after incubation.

### 2.3. Cytotoxicity

L929, a mouse fibroblast cell line, was obtained from the America Type Culture Collection. RPMI 1640 (HyClone, Logan, UT, USA) was used to culture the cells and was supplemented with 10% FBS and 1% penicillin and streptomycin. CA was diluted with medium for the assay, and the same amount of DMSO (0.1%) was added to the negative control. According to the manufacturer’s protocol, a Cell Counting Kit-8 (CCK-8, Dojindo Molecular Technologies, Kumamoto, Japan) was used to assess cytotoxicity.

### 2.4. Oral Gavage of the P. gingivalis Mouse Model

Eleven-week-old female BALB/c mice were purchased from Samtako Bio Korea (Osan, Korea). This experiment was approved by the Wonkwang University Animal Ethics Committee (WKU20-97). The mice were randomly divided into five groups. Eight animals were assigned per group, and four mice were housed per cage. The experimental groups were divided, and the code, explanation, and treatment were specified in the parenthesis as follows:

Group 1: Negative control (NC; no bacterial inoculation, no treatment)
Group 2: Positive control (PC; bacterial inoculation, no treatment)
Group 3: CA in drinking water (WCA; bacterial inoculation, 0.5% CA extract in drinking water)
Group 4: Varnish (V; bacterial inoculation, varnish)
Group 5: CA varnish (VCA; bacterial inoculation, varnish mixed with CA)

**Figure 1:**
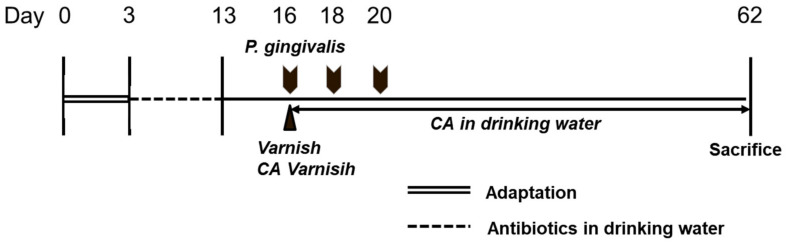
Experimental design. An adaptation period of 3 days was provided, followed by antibiotic drinking water for 10 days. After 3 antibiotic-free days, Varnish and *Colocasia antiquorum* var. *esculenta* (CA) varnish were applied to the teeth, and *P. gingivalis* was inoculated. CA in the drinking water group was provided as distilled water with 0.5 *w*/*v*% CA extract. After 62 days, the animals were sacrificed.

The experimental schedule is shown in Figure 1. All mice were subjected to three days of acclimatization and received deionized water and food ad libitum. After the adaptation period, the mice received sulfamethoxazole (870 μg/mL) and trimethoprim (170 μg/mL) in deionized water for 10 days. Three days after antibiotic treatment, log-phase broth *P. gingivalis* was centrifuged and suspended to make 1 × 10^10^ CFU/mL in 2% carboxymethyl cellulose in PBS. *P. gingivalis* (100 μL/mouse) was inoculated by oral gavage three times every two days in Groups 2, 3, 4, and 5. Three-quarters of *P. gingivalis* was injected into the esophagus using an oral zonde (20G, JEUNGDO BIO PLANT Co., Ltd., Seoul, Korea), while the rest was injected into the oral cavity.

For the WCA group, distilled water mixed with 0.5 *w*/*v*% CA dissolved in 1% ethanol solution was provided as drinking water. Varnish was fabricated with rosin and ethanol as described in the previous study [16]. For the VCA group, 15 wt% CA was mixed into the varnish. For the V and VCA groups, the manufactured varnish and the varnish combined with CA were applied around the upper molar with a micro-brush for 15 s under anesthesia with intraperitoneal injections of 400 mg/kg chloral hydrate (Sigma, St. Louis, MO, USA). All mice were euthanized by CO_2_ gas 42 days after the final bacteria inoculation.

### 2.5. Oral Microbiome

**Figure 2:**
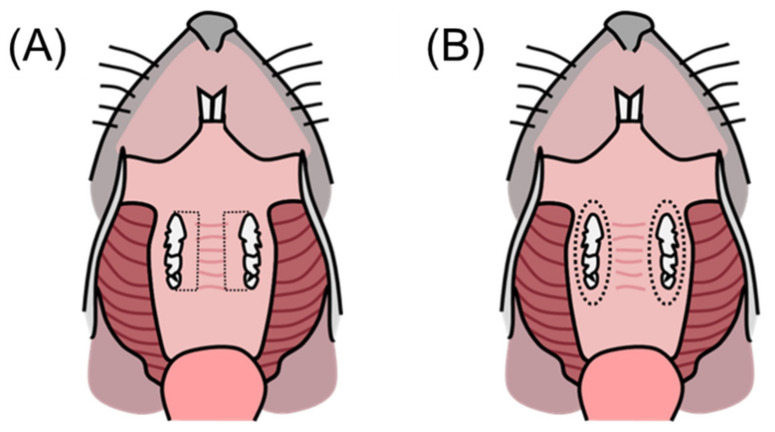
Schematic view of the gingival tissue collection area in the upper jaw of the mouse as expressed with a dotted line. (**A**) Gingival tissue for oral microbiome analysis, (**B**) gingival tissue around the maxillary molar for quantitative real-time PCR analysis.

The experimental animals assigned eight per group were divided into two subgroups. Half of the mice (*n* = 4) were utilized for oral microbiome analysis, while the other four mice were used for inflammatory cytokine analysis. Mouse gingival tissue was collected from the palatal gingiva around the upper molars [30] (Figure 2A). The sample was placed in 500 μL of buffer (Nucleic acid preservation & transport medium, Noblebio, Korea). The collected mouse gingival tissue was transferred into the QIAamp DNA Stool Mini Kit (QIAGEN, Hilden, Germany) solution and the bacterial genomic DNA was extracted according to the manufacturer’s instructions. The library was prepared by referring to Illumina’s 16S metagenomic library prep guide sequencing (Illumina, San Diego, CA, USA). The library was quantified through Qubit4.0 (Thermo Fisher Scientific, Waltham, MA, USA), and the size was measured using Qsep1 (Bioptic Inc., New Taipei, Taiwan). After adjusting the concentration of the prepared library to 20 pmol and pooling to create a mixture, 20 µL of the sample was placed into the cartridge, and analysis was performed using Iseq100 (Illumina) equipment. The FASTQ file created by the Iseq100 equipment was analyzed for its metagenome through the EZBioCloud (ChunLab, Seoul, Korea) platform and the BaseSpace (Illumina, San Diego, CA, USA) platform. After analyzing the samples, the Shannon index, operational taxonomic units (OTUs) counts, and the relative abundances of species, genus, class, and phylum were analyzed. The Shannon index is a well-known diversity index, and the higher the Shannon index value, the higher the community diversity [31]. OTUs are a cluster of similar sequence variants of the 16s rDNA marker gene sequence [32].

### 2.6. mRNA Expression by Quantitative RT-PCR

Real-time polymerase chain reaction (RT-PCR) was performed to analyze the inflammatory factors IL-1β and MMP-9. The mouse gingival tissue collected around the second molar (Figure 2B) was homogenized (BioMasher II; Nippi, Tokyo, Japan) for several minutes in a TRIzol solution (Invitrogen, Carlsbad, CA, USA). cDNA was synthesized from 100 ng of RNA isolated using the Omniscript Reverse Transcriptase Kit (Qiagen, Germantown, MD, USA). Real-time quantitative PCR was analyzed using StepOne Plus Real-Time OCR Systems (Applied Biosystem, Foster City, CA, USA) by adding cDNA synthesized, each with 10 pmol of prime and PowerUp SYBR Green Master Mix (AppliedBiosystem, Waltham, MA, USA). The expression levels of inflammatory cytokine (IL-1β) and MMP-9, as quantified by GAPDH, were compared. Information on the primers used in the experiment is as in Table 1.

### 2.7. Alveolar Bone Loss

For oral microbiome analysis, the skulls were defleshed and stored in 4% paraformaldehyde overnight. Before microCT analysis, paraformaldehyde was replaced with phosphate-buffered saline. The maxilla was scanned with a micro-CT (SkyScan 1076, Bruker, Kontich, Belgium) under 88 kV and 112 μA of X-ray tube voltage and current, respectively; pixel size of 9 μm; and exposure time of 2000 ms at 0.42° intervals. DataViewer software (Bruker, Kontich, Belgium) was used to reconstruct the two-dimensional images, while CTVox (Bruker) was utilized to create a three-dimensional (3D) image. Using Image J software (NIH, Bethesda, MD, USA), alveolar bone loss was measured as the distance from the cementoenamel junction (CEJ) to the alveolar bone crest (ABC) on the 3D images at a total of seven buccal sites [33].

### 2.8. Statistical Analysis

Statistical analysis was performed using IBM SPSS Statistics (Version 26, Armonk, NY, USA). When the data has its normality and homogeneity of variance with the Shapiro–Wilk test and the Levene test, it was analyzed with a one-way analysis of variance test. Cytotoxicity was analyzed using a one-way analysis of variance with Tukey’s multiple range test. Alveolar bone loss data were analyzed using one-way analysis of variance with the post hoc Duncan’s multiple range test. Real-time PCR data were tested with the Kruskal–Wallis test, with the post hoc Mann–Whitney test and the Bonferroni correction. Oral microbiome data were tested by the Kruskal—Wallis test. A *p*-value < 0.05 was considered statistically significant.

## 3. Results

### 3.1. Antibacterial Effect and Cytotoxicity of CA

**Figure 3:**
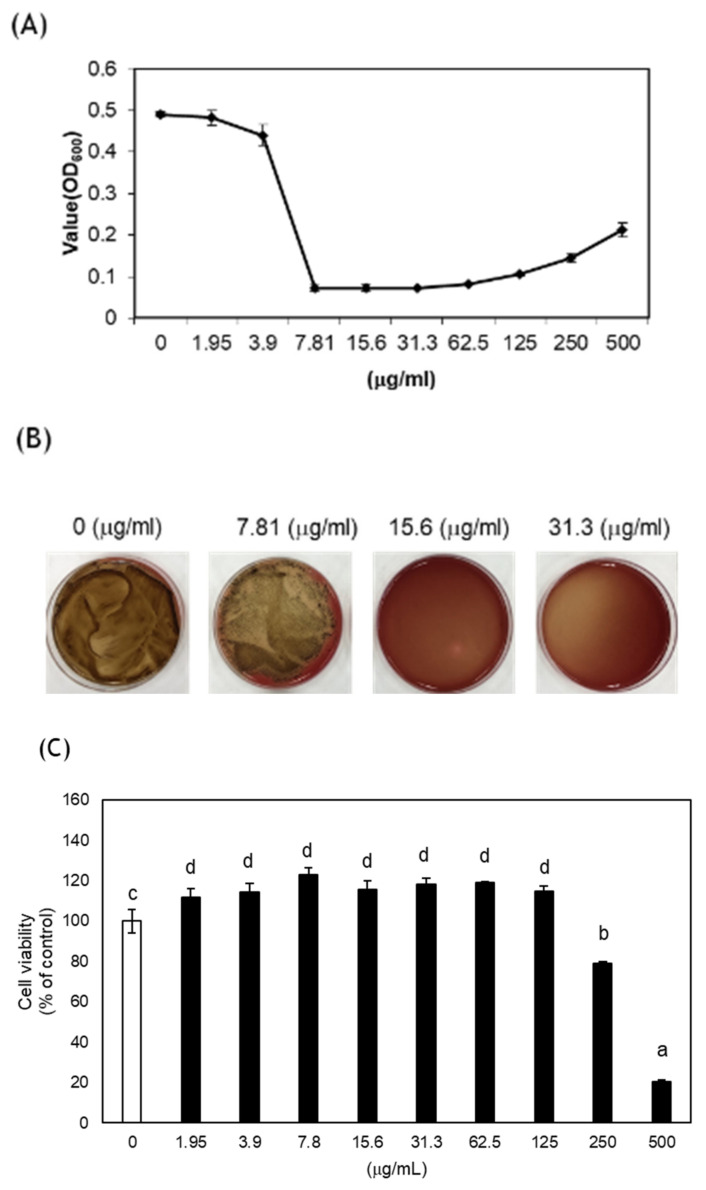
Antibacterial effects and cytotoxicity of *Colocasia antiquorum* var. *esculenta* (CA). (**A**) The minimal inhibitory concentration of CA against *P. gingivalis* ATCC 53978, (**B**) the minimal bactericidal concentration of CA against *P. gingivalis*, (**C**) the cytotoxicity of CA on L929 cells as determined by the CCK-8 assay. Different letters indicate significant differences between the groups by one-way ANOVA with Tukey’s multiple comparisons (*p* < 0.05).

CA significantly inhibited the growth of *P. gingivalis* ATCC 53978 with a minimal inhibitory concentration of 7.81 μg/mL (Figure 3A) and a minimal bactericidal concentration of 15.6 μg/mL (Figure 3B). CA exhibited no cytotoxicity up to the concentration of 125 μg/mL. The cytotoxicity significantly increased at 250 μg/mL or higher concentrations than NC (Figure 3C).

### 3.2. Oral Microbiome

**Figure 4:**
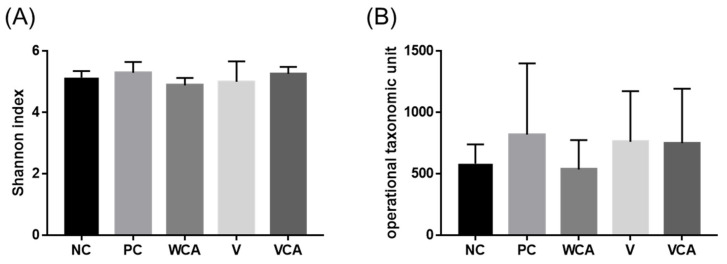
Alpha diversity of the oral microbiome, (**A**) Shannon index of the mouse gingival tissue microbiome. (**B**) Operational taxonomic unit (OTU) of the microbiome. There were no significant differences in alpha diversity. NC, negative control; PC, positive control; WCA, water in *Colocasia antiquorum* var. *esculenta* (CA) group; V, varnish group; VCA, Varnish with CA group.

In the microbiome analysis, a total of 3073 species were analyzed. Alpha diversity was assessed by the Shannon index and operational taxonomic unit (OTU) analysis (Figure 4). There were no statistically significant differences among the groups in Shannon index or OTUs (*p* > 0.05).

**Figure 5:**
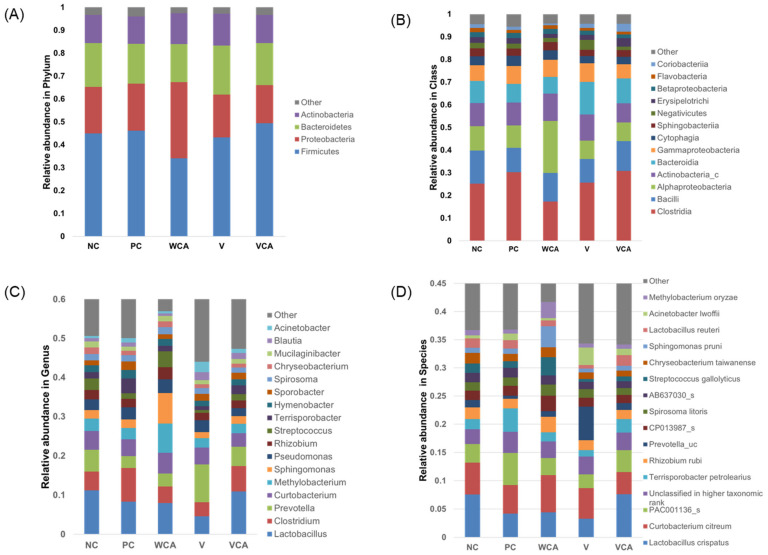
Bacterial taxonomic distributions. (**A**) Phylum level, (**B**) class level, (**C**) genus level, and (**D**) species level. NC, negative control; PC, positive control; WCA, water in *Colocasia antiquorum* var. *esculenta* (CA) group; V, varnish group; VCA, varnish in CA group.

The relative abundance at the phylum, class, genus, and species levels are demonstrated in Figure 5. Bacteria that comprised less than 0.5% of the total bacteria were classified as other. At the phylum level, Firmicutes were the most dominant in all groups. At the class level, Clostridia dominated, followed by Bacilli. At the genus level, Lactobacillus was the most dominant. At the species level, *Lactobacillus crispatus* was the most dominant in the NC and VCA groups.

Bacteria related to periodontitis are summarized in Table 2. The relative abundance of Spirochaetes was smaller in NC than PC, and those of the experimental groups tended to be smaller. The relative abundance of Bacilli, Lactobacillus, and *Lactobacillus crispatus* were larger in NC than PC, and the VCA group exhibited a similar tendency to NC.

### 3.3. Anti-Inflammatory Effect

**Figure 6:**
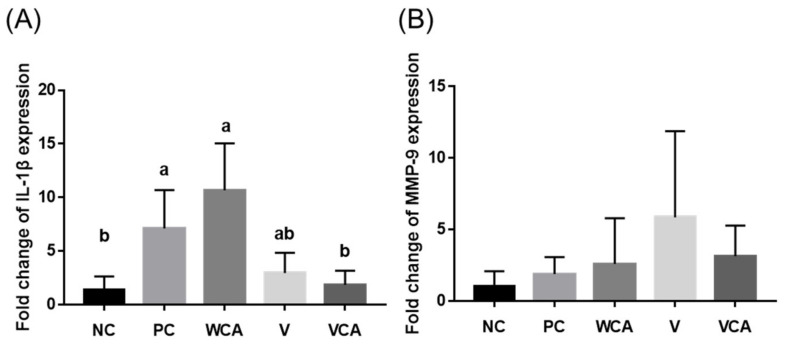
(**A**) Interleukin 1-β and (**B**) matrix metallopeptidase-9 (MMP-9) levels in mouse gingival tissue. Different lowercase letters indicate significant differences by the Kruskal–Wallis and Mann–Whitney U tests with the Bonferroni correction at *α* = 0.05. NC, negative control; PC, positive control; WCA, water in CA group; V, varnish group; VCA, varnish with CA group.

The VCA group showed significantly lower IL-1β expression level than PC and WCA groups (*p* < 0.05), with no significant differences from the NC and V groups (*p* > 0.05) (Figure 6A). There were no statistically significant differences in MMP-9 expression between the groups (Figure 6B).

### 3.4. Alveolar Bone Loss

**Figure 7:**
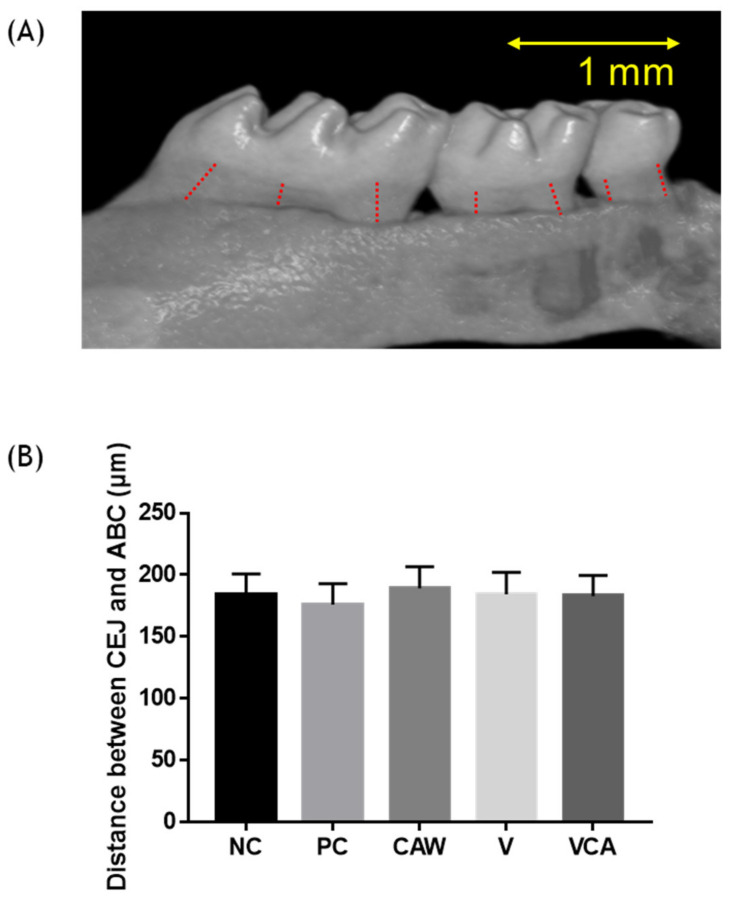
Micro X-ray computed tomography (CT) analysis of alveolar bone loss. (**A**) The micro-CT image shows the distance between the cemento-enamel junction (CEJ) and the alveolar bone crest (ABC) represented by the dotted line. (**B**) Alveolar bone loss is expressed as the measured distance of the CEJ to the ABC of the groups. NC, negative control; PC, positive control; WCA, water in *Colocasia antiquorum* var. *esculenta* (CA) group; V, varnish group; VCA, varnish in CA group.

There were no significant differences in alveolar bone loss, defined as the distance from CEJ to the ABC, between the groups (*p* > 0.05) (Figure 7).

## 4. Discussion

The purpose of this study was to evaluate the effect of CA extract in vitro and CA varnish in vivo in terms of improving periodontitis. The antibacterial effects against *P. gingivalis* and cell cytotoxicity of CA extract were measured in vitro, while the oral microbiome, mRNA expression (IL-1β and MMP-9), and alveolar bone loss were evaluated by oral gavage of the *P. gingivalis* mouse model treated with CA varnish.

For the antibacterial effect of CA extract against *P. gingivalis* ATCC 53978, MIC and MBC were 7.81 and 15.6 μg/mL, respectively, with no cytotoxicity. In a previous study, MIC and MBC against *P. gingivalis* ATCC 33277 were 31.3 and 62.5 μg/mL, respectively [34]. *P. gingivalis* is a Gram-negative anaerobic bacterium and one of the main causes of periodontal disease [35]. Since fimbriae vary according to the substrain of *P. gingivalis*, the response according to the pathogenicity or agent may be different [36]. There are multiple substrains in *P. gingivalis*, and the virulence varies between them [37]. It was confirmed that MIC and MBC were altered according to the substrain of *P. gingivalis*, and higher antibacterial activities of CA extract were demonstrated against *P. gingivalis* ATCC 53978 than *P. gingivalis* ATCC33277.

In another previous study, a methanol extract of CA exhibited antibacterial activity of 125 μg/mL against *P. gingivalis* ATCC 33277 [16]. Although the solvent was different, the antibacterial activity was maintained. Since methanol is neurotoxic, the ethanol extraction is expected to be useful in the future.

The cytotoxicity of CA was not observed up to 125 μg/mL but significantly increased at 250 μg/mL; however, considering that ISO 10993-5 specifies cell viability less than 70% of the NC as cytotoxic [38], a concentration of up to 250 μg/mL is not cytotoxic. It was confirmed that there was no cytotoxicity at the concentration exhibiting effective antibacterial activity. It was shown that CA extract is a potential substance for preventing periodontitis in vitro.

For the oral microbiome analysis, oral swabs or gingival tissues were used [39]. The dominant species were clearly visible in the oral swab method, while the results were more varied in the tissue analysis [29]. Since the microbiome in the oral cavity varies by region, oral swabs might be advantageous for obtaining overall microbiome data; however, in order to evaluate periodontitis-related bacteria, evaluation of the gingival tissue around the gingival sulcus is more critical than the overall bacteria of the oral cavity [26]. Therefore, sectioned gingival tissue was used to analyze the microbiome in this study. As a result of the animal experiments, overall microbiome dysbiosis was not observed. There were no significant differences in the Shannon index and OTU, indicating diversity within the group. Antibiotics and chlorhexidine mouthwash used to control the periodontal disease can alter the oral microbiome to cause side effects [40,41]. In this respect, CA varnish, which exhibited little effect on the diversity of the microbiome, is considered advantageous.

At the phylum level, Firmicutes, Proteobacteria, Bacteroidetes, and Actinobacteria were found in more than 95% of all groups. Between them, Firmicutes appeared to be dominant and were mostly Gram-positive. In addition, Spirochaete, which is known to increase in periodontitis [42], tended to increase in PC compared with the NC group and decreased in the experimental groups (Table 2). Bacilli are known to decrease in proportion to the presence of periodontitis [43]. Bacilli at the class level decreased in PC and increased in the VCA group as in the NC group (Table 2).

At the genus level, Lactobacillus was dominant. Lactobacillus plays an important role in maintaining healthy conditions by stimulating natural immunity and contributing to the balance of microflora [44]. In VCA group, Lactobacillus was as dominant, as in NC group. However, Lactobacilli, which can appear in the oral cavity of human beings such as *L. acidophilus* and *L. casei*, were hardly found in mice gingival tissue [45]. At the species level, *Lactobacillus crispatus* represents the majority of Lactobacilli and has not been well studied in the human oral cavity. *Lactobacillus crispatus* tended to increase in VCA similar to NC. *Lactobacillus crispatus* inoculation was reported to improve periodontitis [46]. According to the study of the mouse microbiome with oral swabs, *Streprococcus danieliae* was the dominant species [33]. Although statistical differences were not noted among the bacterial taxonomic distributions due to the limited number of specimens, a tendency to change in the microbiome composition was observed.

Alveolar bone loss analyzed using micro-CT showed no statistically significant differences between the groups. Possible reasons why are as follows. Firstly, the number and amount (CFU) of inoculations were not enough to cause periodontitis. Although there have been several inoculation doses in several inoculation models [47,48], three inoculations were performed in this study, following the protocol of PJ Baker [49,50]. Secondly, several studies have adopted periodontitis models of mixed infection with *P. gingivalis* and other bacteria such as *Fusobacterium nucleatum* or *Streptococcus gordonii* [51,52]. The previous study was performed using *P. gingivalis* ATCC 33277 [34]. According to Baker’s research, *P. gingivalis* ATCC 53978 exhibited more potent virulence and bone loss than ATCC 33277 [53]. Therefore, *P. gingivalis* ATCC 53978 was used to induce alveolar bone loss in this study. In this study, *P. gingivalis* single infection did not cause alveolar bone loss, despite using *P. gingivalis* ATCC 53978, which is known to have higher virulence [52]. Thirdly, instead of using bacteria grown on plates, the bacteria were grown in broth. Therefore, there is a high possibility of inducing bone loss, because bacteria with black pigmentation on the plate are more toxic than those grown in broth [54]. Further research is needed to evoke alveolar bone loss by adjusting for these factors.

The expression of IL-1β of the gingival tissue was higher in PC than NC and decreased in the VCA group. IL-1β is one of the pro-inflammatory cytokines observed in the early stages of inflammation [55]. MMP-9 is induced by various inflammatory stimuli and associated with bone destruction [56]; however, MMP-9 did not exhibit a statistically significant difference between groups, which is consistent with those of alveolar bone loss. Although bone destruction was not induced in this study, significant changes in the pro-inflammatory cytokine had occurred. Therefore, it is considered that slight inflammation such as gingivitis had occurred but had not progressed to periodontitis. It is necessary to increase the concentration of the CA extract for further study.

The WCA group was included to evaluate the systemic effect of CA by adding it to drinking water; however, a higher level of IL-1β was observed in the WCA group, where CA was metabolized and circulated throughout the body. The concentration used in this study seemed not to be strong enough to exhibit an anti-inflammatory effect. If CA is administered through oral gavage directly at higher and consistent concentrations, it is expected to demonstrate more reliable systemic anti-inflammatory effects.

In this study, it was not possible to verify which compounds in CA extracts were effective for antibacterial and anti-inflammatory activities. In addition, when mixed with varnish, the release profile of the extracts was not evaluated. It is necessary to analyze the active substances with HPCL or LC-MC. For inflammatory factors, future studies on other cytokines that cause inflammation and bone loss are needed [57,58]. For microbiome analysis, increasing the number of animals in each group or analysis using oral swabs seems to be necessary for further studies.

Our research suggests that CA extracts have an antibacterial effect without cytotoxicity at active concentrations in vitro. Using oral gavage of the *P. gingivalis* mouse model, alveolar bone loss was not observed. However, pro-inflammatory cytokine levels were decreased in the CA varnish group. Although there were no significant changes in the overall oral microbiome, there were tendencies to increase the levels of bacteria known to inhibit periodontitis and decrease the levels of bacteria-associated periodontitis in the CA varnish group.

## 5. Conclusions

Within the limitations of this study, CA exhibited antibacterial activity against *P. gingivalis* ATCC 53978 and revealed no cytotoxicity at a concentration that showed antibacterial activity. CA varnish did not change the oral microbiome and alveolar bone loss but showed an anti-inflammatory effect in the oral gavage of the *P. gingivalis* mouse model. CA varnish has the potential to be a suitable candidate for improving periodontitis.

## Figures and Tables

**Table 1:** Sequence of the primers used for quantitative real-time PCR in the study.

Primers	Forward	Reverse
GAPDH	AGGTTGTCTCCTGCGACTTCA	CTGTTGCTGTAGCCGTATTCATTG
IL-β	CTATACCTGTCCTGTGTAATGAAAGA	TCTGCTTGTGAGGTGCTGATGTA
MMP-9	CCCTGGAACTCACACGACATC	GTCCACCTGGTTCACCTCATG

**Table 2:** The relative abundance of bacteria associated with periodontitis expressed as mean and standard deviation in parentheses.

		NC	PC	WCA	V	VCA
Phyla	*Spirochaetes*	0.724 (0.488)	1.438 (1.550)	0.628 (0.428)	0.555 (0.478)	0.930 (1.031)
Class	*Bacilli*	14.554 (8.278)	10.784 (2.647)	12.632 (7.476)	10.409 (6.532)	13.187 (6.558)
Genus	*Lactobacillus*	11.206 (8.017)	8.333 (2.527)	7.986 (3.252)	4.592 (2.774)	10.912 (6.546)
Species	*Lactobacillus crispatus*	6.529 (4.981)	3.585 (1.580)	3.539 (2.593)	2.648 (1.689)	6.632 (4.537)

NC, negative control; PC, positive control; WCA, water in *Colocasia antiquorum* var. *esculenta* (CA) group; V, varnish group; VCA, Varnish with CA group.

## Data Availability

The data presented in this study are available on request from the corresponding author.
